# Human bronchial air-liquid interface responses to nebulised protease exposure

**DOI:** 10.3389/fimmu.2026.1875147

**Published:** 2026-08-07

**Authors:** Georgina Hopkins, David Sheffield, Hugh Barlow, Anja Lalljie, Victoria James, Stella Cochrane, Lucy C. Fairclough

**Affiliations:** 1 School of Life Sciences, The University of Nottingham, Nottingham, United Kingdom; 2 School of Medicine, Pharmacy, and Biosciences, Aston University, Birmingham, United Kingdom; 3 Safety, Environmental and Regulatory Science, Unilever, Sharnbrook, Bedfordshire, United Kingdom; 4 School of Veterinary Medicine and Science, The University of Nottingham, Nottingham, United Kingdom

**Keywords:** air liquid interface (ALI), extracellular vesicles (EV), primary epithelial cells, protease, transcriptomics

## Abstract

**Background:**

As part of a programme developing human-relevant new approach methodologies (NAMs) for next-generation risk assessment (NGRA), we utilised a human air-liquid interface (ALI) bronchial epithelial model (MucilAir™) to assess epithelial responses to nebulised protease exposure.

**Methods:**

Protease doses of 0.005, 0.08, 2, and 24 µg/cm^2^ were applied to MucilAir™ inserts via nebulisation using a Vitrocell^®^ Cloud Alpha. Effects on epithelial barrier integrity, cytokine release, extracellular vesicle (EV) profiles, and EV transcriptomic cargo were evaluated.

**Results:**

Phosphate-buffered saline (PBS) nebulisation produced no detectable barrier effects using the methods described. Protease doses of 2 µg/cm^2^ or lower caused no significant responses compared with PBS controls. Protease doses of 24 µg/cm^2^ induced reductions in TEER, GRO-α, IL-8/CXCL8, IL-9, and VEGF production, but increased MIF production, and EV alterations, indicating barrier disruption. Transcriptomic analysis of MucilAir™-derived EVs indicates a protease dose-dependent increase in differentially expressed microRNAs (miRNAs) that were enriched for pathways involved in epithelial signalling, immune and inflammatory regulation, cytoskeletal organisation, and cell survival and repair.

**Discussion:**

These findings demonstrate the suitability of nebulised dosing for assessing airborne protease effects in bronchial ALI cultures. These data also highlight EV-miRNA profiling as a powerful, non-invasive readout that captures early molecular perturbations not detectable by conventional functional assays alone. Through identification of TEER, EV, and cytokine disruption thresholds to protease through statistical modelling, this work strengthens the methodological basis for generating NAM-aligned *in vitro* data to support risk assessment of inhaled enzymes and other airborne materials.

## Introduction

New approach methodologies (NAMs) are being developed for safety assessments that are more mechanism-focused and human-relevant, including *in vitro* techniques for measuring the effects of chemical substances on the human respiratory and immune systems (1–6).

Proteases, commonly used in household and industrial cleaning products, are recognised respiratory sensitisers and can lead to occupational asthma in the absence of adequate exposure controls (7–10). Previously, we demonstrated the use of an organotypic multi-cell type bronchial epithelial model, grown at the air-liquid interface (ALI) (MucilAir™ model) to measure protease enzyme effects *in vitro* (11). This model consists of a pseudostratified epithelium which closely mimics *in vivo* conditions and has been demonstrated to reflect the physiological conditions in the upper airways (12, 13). To ensure relevance across a broad exposure spectrum, protease deposition was contextualised using inhalation dosimetry approaches, including the Multiple-Path Particle Dosimetry (MPPD) model, and protease solutions were subsequently added in liquid-form to the apical side of the MucilAir™ model, with 4 repeated doses over 8 hours. Despite being a robust model, our previous work (11), together with existing literature (14), indicated that repeated liquid-based delivery of chemical substances alters epithelial cell phenotype, inducing the release of pro-inflammatory cytokines such as interleukin-8 (IL-8)/CXCL8, even when cells are exposed to vehicle controls alone, with these effects dependent on volume and duration of exposure. These findings underscore a key limitation of repeated liquid exposure models for *in vitro* lung risk assessment, as the delivery method itself can provoke non-physiological inflammatory responses. Aerosolised delivery approaches may minimise this artefactual stimulation and more closely recapitulate physiologically relevant airway exposure conditions. Hence, the current work adopts an aerosolised approach to deliver protease to the MucilAir™ model.

In addition, this study focuses on measuring extracellular vesicles (EVs), released by the bronchial epithelial cells, for incorporation into enzyme risk assessments. Bronchial epithelial cell-derived EVs are increasingly recognised as mechanistically relevant mediators of airway barrier integrity and immune modulation in allergy (15–18), aligning with key early events within adverse outcome pathways (AOPs) for respiratory toxicity (19). These EVs express surface tetraspanin proteins, such as CD9, CD63, and CD81, and carry a diverse cargo of proteins, lipids, and nucleic acids, including microRNAs (miRNAs), which can change upon chemical exposure and subsequently alter respiratory responses (20, 21). For instance, in human bronchial epithelial cells, EV-associated miRNAs such as miR-21, miR-142a, and members of the miR-200 family have been implicated in processes directly linked to AOP molecular and cellular key events, including tight junction disruption, epithelial remodelling, and inflammatory signalling (22, 23). Thus, following exposure to chemical substances, changes in EV release and EV-miRNA composition may represent an early molecular initiating event or downstream key event, propagating stress and inflammatory signals to neighbouring epithelial cells and resident immune cells. Incorporating EV profiling into *in vitro* respiratory risk assessment frameworks offers a more sensitive endpoint that complements traditional cytotoxicity and cytokine readouts, capturing early effects that may not be detected by traditional assays.

The present study aimed to evaluate the impact of nebulised protease exposure on human upper airway epithelial integrity and EV-mediated signalling using the MucilAir™ model. In contrast to prior work employing repeated apical liquid application of protease to simulate repeated occupational exposures (11), this study introduces an aerosol delivery system capable of depositing protease in a manner more biologically relevant. As no effects were seen previously at protease exposures relevant to human exposures, this study focuses on using higher concentrations to identify an *in vitro* threshold for epithelial disruption and compare the impact of the different dosing approaches. These higher concentrations were selected to define the onset of adverse epithelial responses rather than to replicate typical workplace exposures. Following nebulised delivery, epithelial barrier function, cytokine release, and EV production were assessed, with a particular focus on EV-derived miRNA transcriptomics to characterise early, non-invasive biomarkers and mechanistic pathways linked to protease-induced epithelial responses.

Together, this work advances the development of human-relevant *in vitro* approaches for evaluating inhaled protease exposures and identifies EV-miRNAs as sensitive reporters of epithelial disruption and immune-modulatory signalling in response to aerosolised proteases.

## Materials and methods

### Donor characteristics

MucilAir™-bronchial epithelial samples from 3 male, healthy, non-smoker donors were utilised in this study. The characteristics of these donors are shown in **Table 1**. The TEER and cilia beating frequency measurements were obtained from the supplier before shipping the cells.

**Table 1:** Donor characteristics of MucilAir™-bronchial epithelial samples. .

Donor	Age	Sex	Origin	TEER before arrival in lab (Ω.cm2)	Cilia beating frequency (Hz) before arrival in lab	Time from ALI formation to experiment (days)
MD0858	22	Male	Caucasian	346 +/- 9	8.1 +/- 0.4	71
MD0937	29	Male	Caucasian	478 +/- 11	10.9 +/- 0.3	64
MD571	35	Male	Unknown	523 +/- 16	8.9 +/- 0.1	64

### Culture of primary bronchial epithelial cells

The MucilAir™-bronchial (Cat no. EP01MD) samples were purchased from Epithelix Sárl, and each were a fully differentiated bronchial epithelial model reconstituted from primary human cells taken from the donors described. The samples arrived in the format of 24-well transwell inserts (0.33cm^2^), which were then placed in a sterile 24-well plate with 700 µL of phenol red-free MucilAir™ culture medium (Cat no. EP06MM, Epithelix Sárl) placed in the basolateral compartment and then transferred to a humidified incubator (37 °C; 5% CO_2_). The culture medium was changed every 2–3 days.

### Nebulised protease exposure approach

An apical wash with phosphate-buffered saline (PBS) without Ca^2+^/Mg^2+^ (Cat no. D8537, Sigma) was performed on the MucilAir™ insert 4 days prior to any exposure experiment, as per manufacturer instructions, to mimic a normal amount of mucus build-up.

On the day of the exposures, the protease solution (cat no. P3111, Merck), which is a serine protease secreted by a Bacillus sp. (Savinase^®^ 16.0L), was diluted to the desired range of concentrations in sterile PBS (Cat no. D8527, Merck) and stored at 4 °C. Solutions were brought to room temperature before the exposure. In the certificate of analysis, the proteolytic activity of the stock protease solution was given as 17 KNPU/G. A random order generator (URL: https://www.random.org/lists/) was used to determine the plate layout for each experiment. Triplicate wells were utilised for each protease concentration exposure.

Building upon previous work (11) which involved exposing MucilAir™ inserts to liquid protease, we chose a protease concentration range to identify a threshold for significant barrier disruption. Thus, we utilised concentrations of 0.005, 0.08, 2, and 24 µg/cm^2^, corresponding to 4 previous repeated apical liquid exposures of 0.031, 0.5, 12.5, and 150 µg/mL, respectively (**Table 2**). These µg/cm^2^ concentrations were calculated using the total protease mass deposited per insert in the previous study (µg/insert) which was then converted to a surface-normalised deposition (µg/cm^2^) by dividing by the insert surface area. The deposition factor of protease (35) was determined by measuring the amount of enzymatically active protease in wells of PBS after placing in the chamber with a known amount of nebulised protease. The target deposition per insert could then be used in the following calculation to determine how much protease solution to add to the nebuliser (35):

**Table 2:** Nebuliser concentration calculations.

Previous liquid protease concs. (µg/mL)	Previous liquid protease deposition/insert (µg)	Nebulised target deposition (µg/cm^2^)	Stock concentration in nebuliser (µg/mL)
0.03125	0.0015625	0.005	9.326
0.5	0.025	0.078	149.211
12.5	0.625	1.953	3730.283
150	7.5	23.638	44763.393

Using the total protease deposited in previous repeated apical liquid experiments (target dep/insert), this could be used to determine the target deposition in µg/cm^2^ and subsequently the stock concentration of protease needed to add to the nebuliser.


$$Concentration\;of\;stock\;solution(µg/mL)=(Target\;deposition(µg/cm^{2})*Surface\;area\;of\;chamber(200.54\;cm^{2})/Volume\;in\;nebuliser(0.03\;mL))*100\;/Deposition\;factor$$

The nebuliser exposures were conducted using a Vitrocell^®^ Cloud Alpha ax12 system chamber (Vitrocell). The nebuliser and chamber were cleaned with 70% ethanol (v/v) between each exposure. Filtered PBS was added to the nebuliser and activated until empty to rinse the nebuliser before use.

Basal supernatants were harvested for 0 hours (before exposure) analysis, and 500 μL of fresh media was added to the basal compartment of each MucilAir™ insert. Cell inserts were split into groups of 3 technical replicates for each exposure. Then, each protease solution was placed into the nebuliser reservoir for aerosol material exposure. MucilAir™ inserts were individually removed from their culture plates and added to a base plate in the cloud chamber. The nebuliser was then activated until the solution was consumed and allowed to fully gravitationally deposit out of the Cloud Alpha ax12 and onto the plate of MucilAir™ inserts (~ 7 minutes). After exposure, the MucilAir™ inserts were placed back into their culture plates containing the same medium and returned to the incubator for 24 hours.

**Figure 1:**
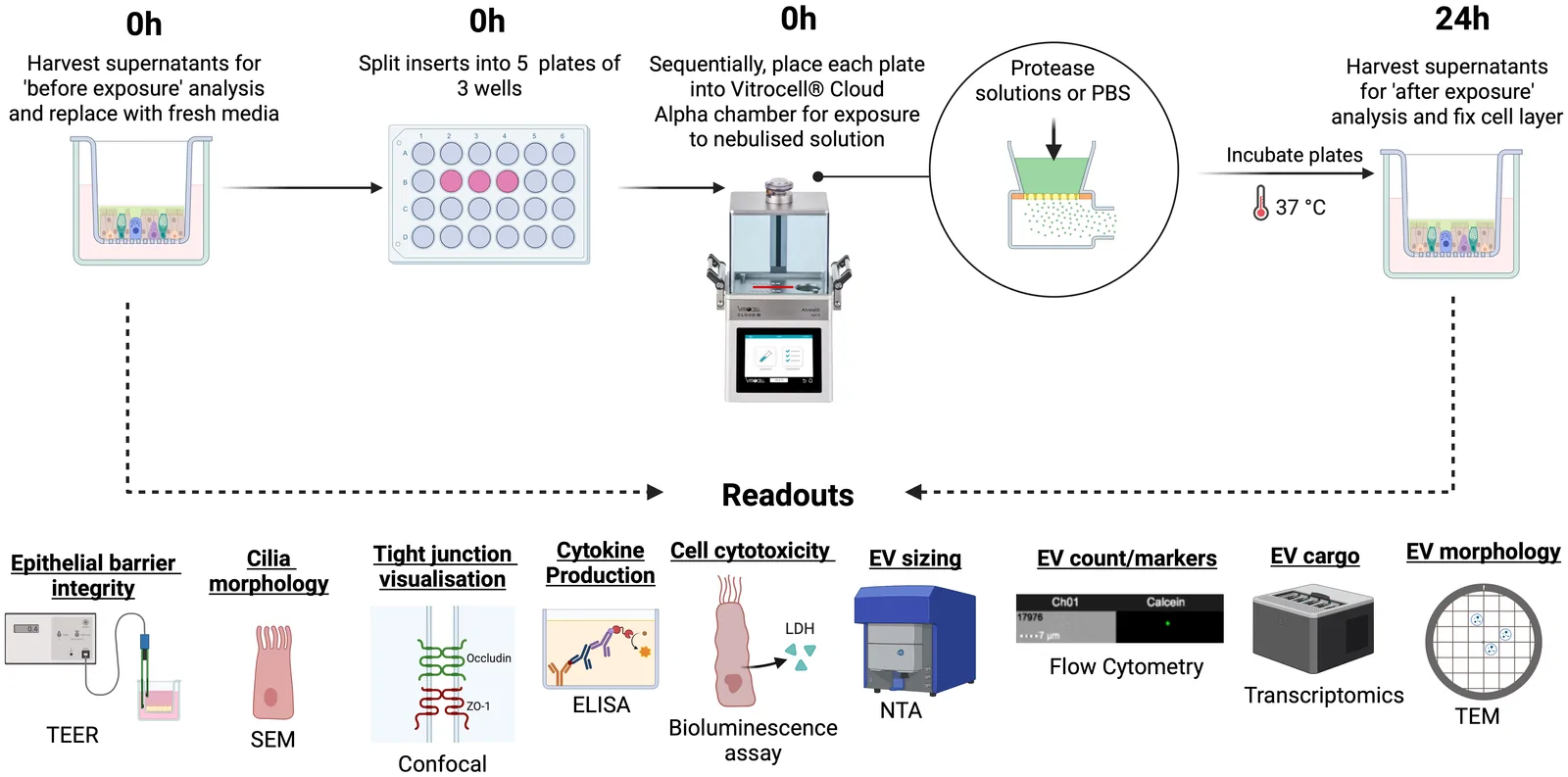
Experimental outline. Basal supernatants at 0h are harvested and stored for analysis and replaced with fresh media. Inserts are then placed into the Vitrocell^®^ Cloud Alpha ax12 system chamber in triplicate, in a fresh plate. Commercially supplied protease is diluted to the desired concentrations and applied to the nebuliser, or in the case of controls filtered PBS applied to the nebuliser. The nebuliser is activated and fully emptied before leaving the particles to settle. The MucilAir™ inserts are then replaced back into their cell culture plate with the same media and incubated for 24h. This is repeated for each control (PBS only) or protease exposure. 24h after the first dose of PBS alone or protease, the epithelial cells and their supernatants are then harvested and analysed for epithelial barrier disruption and immune responses. TEER measurements, scanning electron microscopy (SEM) and confocal microscopy were utilised to measure epithelial barrier integrity, cilia morphology, and tight junction integrity. ELISAs were conducted to measure cytokine responses. A bioluminescence assay for LDH quantification was used to assess cell cytotoxicity. EVs were characterised by flow cytometry (ImageStreamX MKII), and nanoparticle tracking analysis (NTA) for sizing. EVs were also isolated from supernatants, concentrated, and stored for transcriptomics analysis of EV cargo and transmission electron microscopy (TEM). Created using BioRender.

The nebuliser exposure protocol is illustrated in **Figure 1**.

### Apical liquid exposure approach

The results of nebulised PBS exposures were compared to repeated liquid PBS exposures from our previous study (11). For apical liquid PBS exposures, a sterile PBS apical wash was performed on the MucilAir™ insert 4 days prior, as suggested by the manufacturer, to mimic a normal amount of mucus build-up. Triplicate wells were utilised from 7 healthy, non-smoker, male donors under 40 years old. Aliquots (50 μL) of sterile filtered PBS was applied apically to the epithelial transwell insert four times over an 8-hour period and replaced in the incubator at 37 °C; 5% CO_2_ each time. This was to mimic clearance over a prolonged exposure scenario. Before each application, any residual PBS solution from the previous application was removed. After 8 hours, the final PBS solutions were removed from the apical surface, and the samples were incubated at 37 °C; 5% CO_2_ for a further 16 hours.

### Transepithelial electrical resistance measurement

To determine the integrity of the epithelial barrier, TEER measurements were conducted before PBS or protease exposure, and 24 hours after exposure to PBS or protease. Cell Inserts were transferred into wells containing 750 µL of room temperature PBS and an aliquot of 300 µL PBS was also added to each apical chamber. The inserts were left to equilibrate for 10 minutes. TEER was then measured using a Millicell-ERS voltohmmeter with chopstick electrodes (Cat no. MERS00002, Merck), rinsing the electrode in sterile PBS between measurements. The cells were out of the incubator for a total of 20 minutes before aspirating apical and basal solutions and adding 500 µL of fresh MucilAir media to the basal compartment. The voltohmmeter was calibrated before each experiment, using a STX04 test electrode (Cat no. MERSSTX04, Merck), which reads 1000 ohms once calibrated. The electrode was sterilised in 70% ethanol for 10 minutes and rinsed in sterile PBS before use. Values were adjusted for the resistance of the MucilAir™ support membrane (corrected value = measured value − blank insert value), then adjusted for the tissue surface area (0.33 cm^2^).


$$\text{TEER}(\Omega {\text{ cm}}^{2})=\text{Blank}-\text{adjusted resistance reading}(Ω)\times \text{Surface area of insert}({\text{cm}}^{2})$$

### Immunofluorescence staining

After supernatants were harvested, the epithelial cells within transwells were washed four times with PBS; 200 µL apically and 750 µL basally. Cells were then fixed with 4% paraformaldehyde (v/v) (Cat no. 420801, Biolegend), 200 µL applied apically and 750 µL basally, for 30 minutes at room temperature. The fixation buffer was then washed off with PBS and the cells were stored in 1 mL of PBS at 4 °C for up to 1 week. The samples were then permeabilised with 200 µL into apical and 750 µL into basal compartments of 0.05% Triton X-100 (v/v) in PBS for 30 minutes at 4 °C. The cells were washed with PBS three times and then blocked for 1.5 hours with 100 µL of immunofluorescence buffer (PBS containing 5% BSA (Cat no. A7284, Merck)), 0.2% Triton-X (v/v) (Cat no. T8532, Merck), and 0.05% Tween20 (v/v) (Cat no. P7949, Merck)) in the apical chamber. The blocking buffer was removed and cells were incubated with 50 µL of 5 μg/mL AF488–labelled anti-occludin mAb (Lot no. YL381103, Cat no. 10073504, Invitrogen) and 5 μg/mL anti-zonula occludin 1 (ZO-1)–AF647 mAB (Lot no. ZG393516, Cat no. 15648028, Invitrogen), applied apically, overnight at 4 °C in the dark. The cells were then washed with immunofluorescence buffer and removed from the transwell with a scalpel. The cells were then stored in a 24 well plate with 1 mL PBS, at 4 °C protected from light for up to 1 week. Specimens were examined under a Zeiss 900 CLSM confocal microscope and analysed in Zen Lite (Zeiss) software.

### Scanning electron microscopy

A separate batch of inserts were fixed with 4% paraformaldehyde (v/v) (Cat no. 420801, Biolegend), 200 µL applied apically and 750 µL basally, for 30 minutes at room temperature. The fixation buffer was then washed off with PBS and the cells were stored in 1 mL of PBS at 4 °C for up to 1 week. The PBS was then removed and inserts were dehydrated by passing through a graded series of 1 mL ethanol (Cat no. 153386F, VWR) dilutions (50, 70, 90, and 100% (v/v) in ultrapure water). Dehydrated samples were then dried using 50 µL of hexamethyldisilazane (Cat no. 440191, SLS) until evaporated and then mounted on an aluminium stub and sputter coated with platinum (Cat no. AGB7341, Agar Scientific), before viewing on a JEOL IT-200 SEM.

### Cytokine ELISAs

Basal MucilAir™ cell supernatants after 24h of exposures were centrifuged at 300g for 8 minutes to remove any cells. The supernatant was then stored at -80 °C until use in ELISAs. The supernatant was thawed and incubated with 0.2mM of Phenylmethylsulfonyl Fluoride (PMSF) (cat no. 93482, Merck) for 1h on a shaker to inactivate any residual proteases in the supernatants which can interfere with the assay. Technical replicate wells for each donor and exposure condition were pooled. A custom Bio-Plex Pro™ Human Cytokine assay (Bio-Rad) was used to quantify cytokine production in epithelial cell supernatants. The custom panel comprised ten analytes: GRO-α (CXCL1), IL-1rα, IL-6, IL-8/CXCL8, IL-9, IP-10, MCP-1/MCAF, MIF, PDGF-ββ, and VEGF. A volume of 150 μL of each supernatant was transferred to a polypropylene PCR plate (Cat no. 10407314, Fisher Scientific). As samples were collected from serum-free culture conditions, bovine serum albumin (BSA) was added to a final concentration of 0.5% to match the concentration provided in the standards. Samples were analysed undiluted (neat). The assay was performed according to the manufacturer’s instructions. Briefly, magnetic capture beads conjugated to analyte-specific antibodies were added to each well of a 96-well plate and washed prior to sample loading. 50 μL of standards, blanks, controls, and samples were added to the appropriate wells and incubated for 30 min at room temperature with shaking (850 rpm). Following washing, biotinylated detection antibodies were added and incubated for a further 30 min. Streptavidin-phycoerythrin (SA-PE) was then added and incubated for 10 min at room temperature. After a final series of washes, beads were resuspended in assay buffer and analysed using a Bio-Plex^®^ multiplex suspension array system.

Cytokine concentrations were calculated from analyte-specific standard curves generated using an eight-point serial dilution of the supplied standards. Data acquisition and analysis were performed using Bio-Plex Manager software. Results are reported as cytokine concentrations (pg/mL).

### Lactate dehydrogenase release assay

A cell viability assay (LDH-Glo Cytotoxicity Assay, Cat no. J2380, Promega) was performed to determine possible cell toxicity. Basal supernatants were stored in 1:10 in LDH buffer ((200mM Tris-HCl (pH 7.3) (Cat no. 41573, SLS), 10% glycerol (w/v) (, 1% BSA (v/v)) (Cat no. A7284, Merck), at -20 °C until use. Samples were then and incubated with 0.2mM of PMSF (Cat no. 93482, Merck)) for 1h on a shaker at room temperature to inactivate any residual proteases in the supernatants which can interfere with the assay. The assay was then performed according to the manufacturer’s instructions. Briefly, 20 μL of sample was added in duplicate to a white opaque 384-well assay plate (Cat no. 3574, SLS) and mixed with 20 μL of detection reagent. The plate was incubated for 1 hour at room temperature before reading luminescence on a GloMax Explorer plate reader (Promega). To calculate percentage cytotoxicity, 50 μL of 10% Triton X-100 (v/v) treatment was added apically to epithelial cells and incubated at 37 °C for 8h at as a positive control for LDH release. Basal supernatants were then collected. The following calculation was then performed:


$$\text{Percent cytotoxicity}=100\times \frac{\left(\text{Experimental release}–\text{medium background}\right)}{\left(\text{Triton}-\text{X release}–\text{medium background}\right)}$$

### Characterisation of EVs by flow cytometry

Supernatants were removed from transwells 24 hours after the first protease dose and centrifuged at 300g for 8 minutes to remove any cells. 40 μL of supernatants were then immediately incubated with 10 μM calcein AM (Cat no. 425201, Biolegend), 2.5μg/mL of tetraspanins CD9 APC (Clone REA1071, Cat no. 130-118-808, RRID: AB_2733406, Miltenyi), CD63 PE (Clone REA1055, Cat no. 130-118-077, RRID: AB_2733833, Miltenyi), and CD81 PEVio615 (Clone REA513, Cat no. 130-131-387, RRID: AB_2928569, Miltenyi) for 1 hour at 37 °C to stain EVs. The samples were then diluted to 200 μL in filtered PBS and run on the ImageStreamX MkII (Amnis).

### Size exclusion chromatography

Before Transmission Electron Microscopy (TEM) and Nanoparticle Tracking Analysis (NTA), cell supernatants were removed from wells and centrifuged at 300g for 8 minutes to remove any contaminating cells. EVs were then isolated by size exclusion chromatography (SEC) using qEV original 35 columns with an automated fraction collector (Izon Science). Fractions 1–7 of 500 μL were collected, pooled, and concentrated by centrifuging at 4000g for 10 minutes using 10 kDa amicon columns (Cat no. UFC801096D, Merck). The result is ~250 μL of EVs in solution, ranging from 35 nm to 350 nm in size.

### Transmission electron microscopy

MucilAir™ EVs from the PBS control exposures were isolated by size exclusion chromatography and placed on carbon-coated 300 Cu mesh transmission electron microscopy (TEM) grids (company) for 15 minutes and then fixed with 2% paraformaldehyde (v/v) for 10 minutes. The grids were washed 5 times with deionised water before incubating with 4% Uranyl Acetate (w/v) for 10 minutes. Excess stain was removed before allowing to dry completely, and then grids were imaged using a Tecnai BioTwin TEM, operating at 80kV, and images were recorded on a Gatan Orius camera.

### Nanoparticle tracking analysis

SEC isolated EVs were diluted 1 in 100 in 0.22 μM filtered PBS and inserted into an Zetaview mono instrument (Analytik) to determine particle size distribution and quantification. Prior to analysis, a 1:10 dilution of 100 nm carboxylated polystyrene (IZON) and a 1:1000 dilution of 200 nm polystyrene (Malvern Panalytical) nanoparticles were used to calibrate the sensitivity of the instrument. Automatic settings were applied for the minimum expected particle size, minimum track length, and blur settings, with 11 positions. Sensitivity was set at 78, gain at 26.88, and temperature set at 21 °C. Data processing and analysis of particle size distribution were performed using ZetaView Software (8.05.16.SP7) (Analytik).

### RNA isolation of EVs

Supernatants from donor MD0858 were harvested 24 hours after exposures were collected and EVs were isolated by SEC. Each EV sample was then frozen at -80 °C, at a concentration of approximately 10^8^ EVs in 200 μL (As determined by NTA), before shipping for miRNA cargo analysis (TAmiRNA). Total RNA was then extracted from EV suspension using the miRNeasy Mini Kit (Cat. 217004, Qiagen). Each sample was treated with 1000 µL Qiazol. Additionally, a synthetic RNA oligonucleotide mix obtained from the miRCURY Spike-In kit (Cat. 339390, Qiagen) was added to each sample at equimolar amounts to monitor RNA extraction efficiency. After incubation at room temperature for 15 minutes, 200 µL chloroform were added to the lysates followed by cooled centrifugation at 12,000g for 15 minutes at 4 °C. Precisely 650 µL of the upper aqueous phase were mixed with 7 µL glycogen (5mg/mL) to enhance precipitation. Samples were transferred to a miRNeasy mini column, and RNA was precipitated with ethanol followed by automated washing with RPE and RWT buffer in a QiaCube liquid handling robot. Finally, total RNA was eluted in 30 µL nuclease free water and stored at -80 °C until further analysis.

### EV small RNA sequencing

For all samples, 4.25 µL of total RNA was used as an input for small RNA sequencing library preparation. To each RNA sample 1 µL of miND spike-in standards (TAmiRNA) were added prior to small RNA library preparation using the RealSeq Biofluids library preparation kit (Cat.600-00048-SOM, RealSeq Biosciences/BioCat). Adapter-ligated libraries were amplified with 20 PCR cycles using barcoded Illumina reverse primers in combination with the Illumina forward primer. Library quality control was performed using an Agilent Small Fragment Kit (Cat. DNF-476-0500). An equimolar pool consisting of all sequencing libraries was prepared, followed by size selection using the BluePippin system (Sage Science) to enrich for small RNA library fragments, and sequenced in single-read mode with 100 cycles on an Illumina NextSeq 2000 P2 Flowcell.

### EV miRNA bioinformatic analysis

Next-generation sequencing (NGS) data was analysed using the miND^®^ analysis pipeline (24). The resulting differentially expressed miRNAs were investigated using Qiagen Ingenuity Pathway Software. This analysis used MicroRNA Target Filter to identify the gene targets of the differentially expressed miRNAs, genes were selected based on experimental evidence from TargetScan, TarBase, miRecords and Ingenuity Knowledge Base. The miRNA-gene pairings were matched based on the directionality of the miRNA abundance and used to perform pathway enrichment analysis.

### Statistical analysis

All statistical analyses were performed using GraphPad Prism v 10.4.1. Data are presented as mean ± standard deviation (SD) unless otherwise stated. Primary airway epithelial inserts were purchased in triplicate for each donor. The three inserts from a given donor originated from the same individual and therefore represent technical replicate cultures. The number of technical replicates (independent inserts) for each donor, and the statistical test utilised, are specified in the corresponding figure legends.

For all data, the technical repeat measurements were averaged and subsequently used for statistical analysis. Repeated-measures one-way ANOVA statistical models or mixed effects analysis with Sidak’s multiple comparisons tests were conducted for TEER, cytokine, and EV measurements to account for donor-donor variability.

Flow cytometry data collected using the ImageStream X MkII was analysed using IDEAS 6.2 software. The data were then imported into Prism software, (GraphPad), for statistical analysis.

For the statistical modelling of TEER, IL-8/CXCL8, and EV data, each measurement from the stimulated samples was compared against the threshold determined from the baseline. Thresholds were set at P25 - 1.5 x IQR (a standard way of determining values that are below expected levels). A logistic regression model was fit with log concentration as covariate with “success” occurring when the value was within the threshold value and “failure” when it was more extreme. Models were fit using the Logistic Procedure in SAS 9.4. The concentration at which there is a certain probability that the response will be below the threshold is determined from this model fit.

## Results

### The effect of nebulised and repeated apical liquid dosing strategies on MucilAir™ responses to PBS

In our previous work (11), repeated apical liquid dosing was utilised; however, observations from those studies suggested that the dosing procedure itself may influence epithelial responses. Therefore, the present study incorporated a nebulisation-based exposure system to minimise perturbation associated with repeated application and removal of apical liquid.

**Figure 2:**
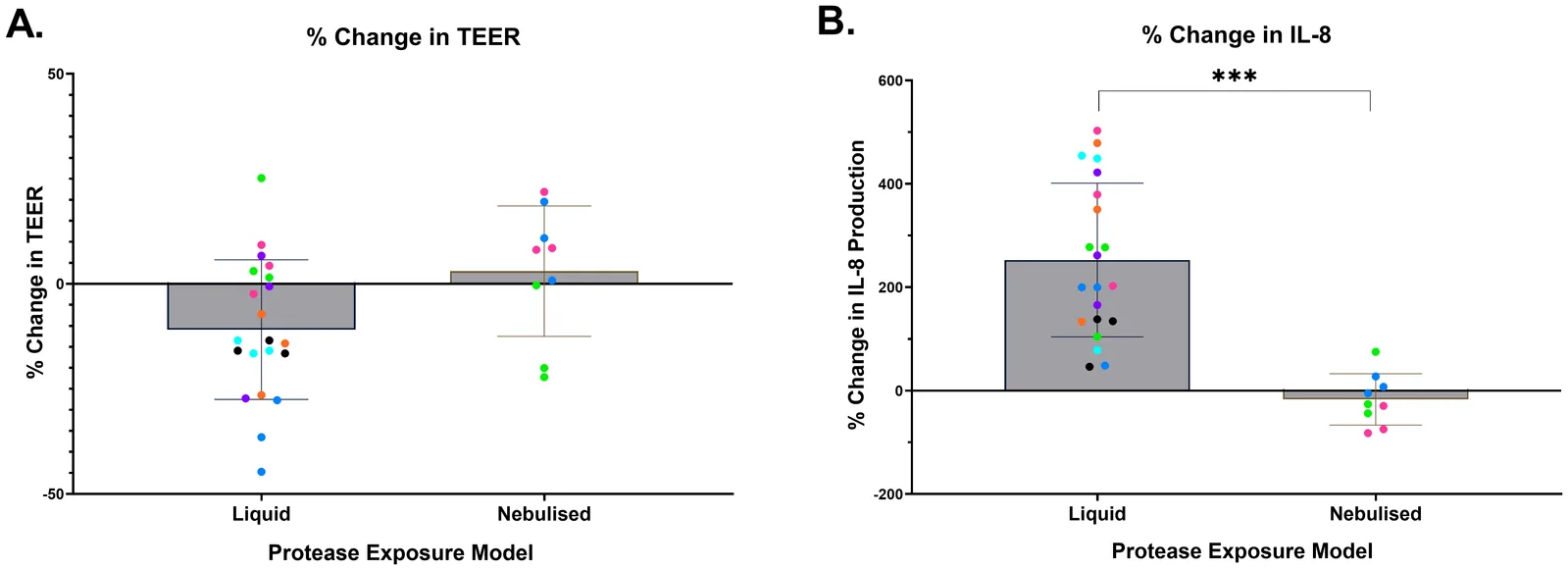
The effect of repeated liquid vs. nebulised dosing with PBS on MucilAir™ readouts. MucilAir™ bronchial epithelial inserts were exposed to repeated apical liquid dosing of PBS once every 2h for a total of 8h, or a single nebulised dose of PBS. The inserts were measured for TEER and IL-8/CXCL8 production before exposures (0h) and 24h after. **(A)** The % change in TEER in each MucilAir™ insert from 0h to 24h of each dosing strategy. **(B)** The % change in IL-8/CXCL8 production from 0h to 24h of each dosing strategy. An unpaired t-test with Welch’s correction was conducted to compare dosing strategies. N = 7 biological donors for the liquid exposure experiments and N = 3 biological donors for the nebulised exposure experiments. For each donor, three replicate inserts derived from the same donor were analysed (technical replicates). ***p<0.001.

Here, data are presented as percentage change from baseline (0 h) to account for inter-donor variability and to facilitate assessment of the impact of the exposure procedure on each donor. Following repeated liquid dosing of PBS, the mean % change in epithelial barrier integrity was comparable to a single nebulised PBS dose (**Figure 2A**). However, cytokine analysis of IL-8/CXCL8 highlighted the mean % change in IL-8/CXCL8 production was significantly higher after the repeated liquid PBS dosing, compared to nebulised PBS dosing (p<0.001) (**Figure 2B**).

### Impact on barrier integrity and cell viability after nebulised protease exposures

Due to no effect of PBS dosing using the nebulised approach, MucilAir™ inserts were then apically exposed to either 0.005, 0.08, 2, or 24 µg/cm^2^ protease, using PBS (0 µg/cm^2^ protease) as a negative control, using the Vitrocell^®^ Cloud Alpha ax12 system chamber. Each donor was exposed to each of these concentrations in triplicate wells.

**Figure 3:**
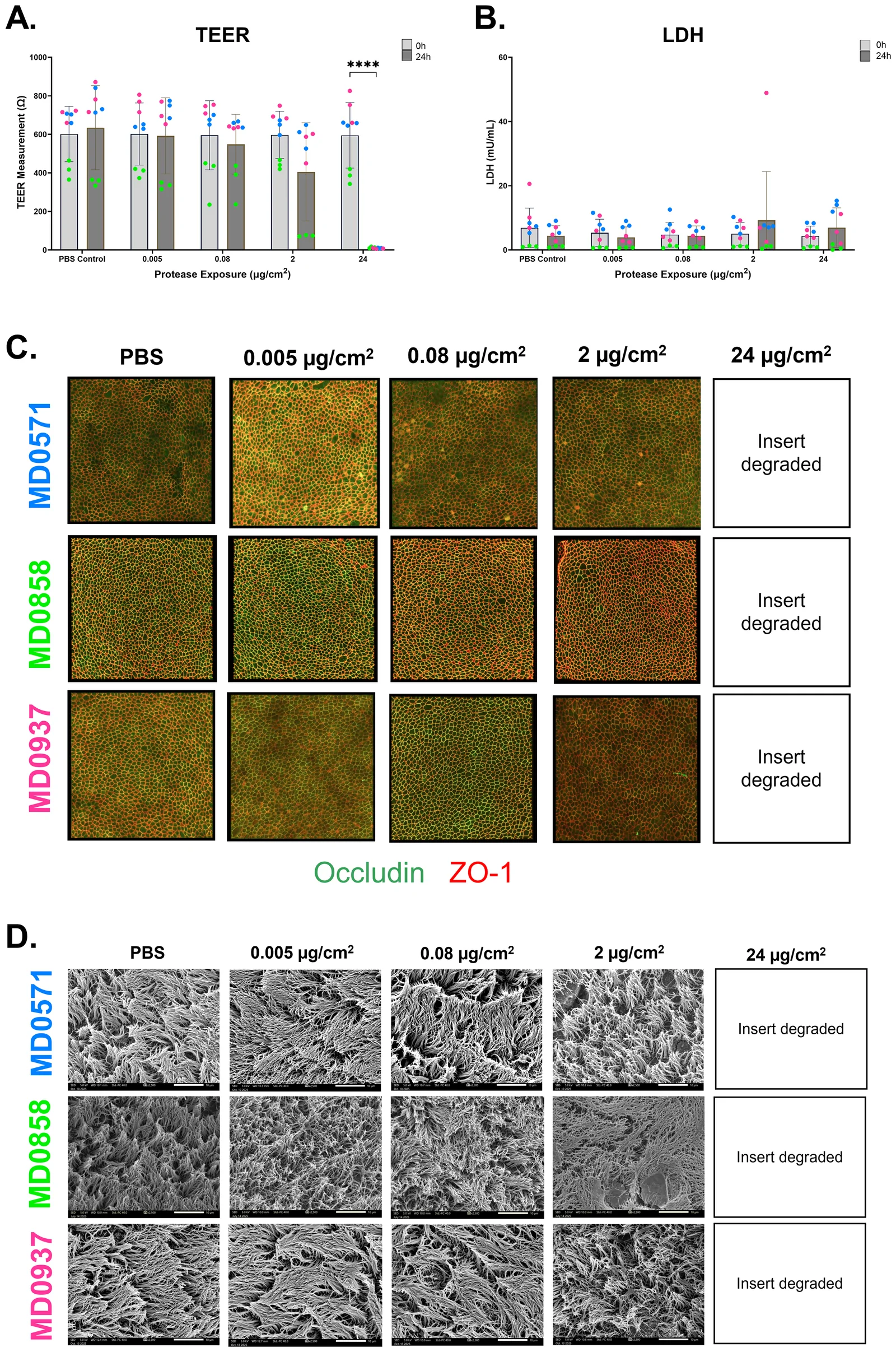
Epithelial barrier integrity. **(A)** MucilAir™ TEER measurements taken before (0h) and 24h after nebulised protease exposures. **(B)** MucilAir™ LDH production before (0h) and 24h after nebulised protease exposures. **(C)** Tight junction microscopy at a magnification of X20, and **(D)** surface morphology of MucilAir inserts exposed to 0 – 24 µg/cm^2^ protease. “Insert degraded” = inserts damaged due to protease and so analysis could not be conducted. A mixed effects model with Sidak’s multiple comparisons was conducted for A-B, ****p <0.0001. N = 3 donors, with 3 replicate inserts measured for each exposure. Each donor is colour coded, MD0571 (blue), MD0858 (green), MD0937 (pink).Cytokine production.

TEER measurements were taken prior to exposure (0 hours) and 24 hours after the exposure (**Figure 3A**). The results show 0-0.08 µg/cm^2^ of protease resulted in no changes to TEER.

measurements, but a trend of decreased TEER measurement was noted after exposure to 2 µg/cm^2^, although not significant. The TEER measurements subsequently significantly decreased at the highest dose of 24 µg/cm^2^ (p<0.0001) nebulised protease.

LDH secretion into the basal supernatants were measured to determine the cell viability of MucilAir™ inserts after protease exposures. PMSF was added to samples 1 hour before conducting the assay to reduce interference of any residual protease in the samples. The results show no significant differences in LDH release (**Figure 3B**). These results were surprising, as some of the inserts visibly degraded (cells were no longer attached to insert and visible) after exposure to 24 µg/cm^2^ of protease. Thus, it was suspected that the protease may degrade LDH over the 24-hour incubation period, before harvesting samples for analysis.

To assess tight junction degradation, 24 hours after exposure, the MucilAir™ inserts were fixed for tight junction staining. Immunofluorescence staining for Occludin and ZO-1 showed no clear evidence of degraded tight junctions for cells exposed up to 2 µg/cm^2^ of protease (**Figure 3C**). Hence, the protease solution does not appear to be affecting tight junction integrity at these concentrations. The MucilAir™ inserts completely detached at the highest protease concentration of 24 µg/cm^2^, thus the immunofluorescence staining could not be conducted.

After confocal microscopy of the tight junctions, a separate batch of inserts were used for cilia morphology visualisation by SEM (**Figure 3D**). Healthy cilia were observed after all exposures, apart from after exposure to 24 µg/cm^2^ protease, where the inserts were again too damaged for analysis.

### Cytokine production

**Figure 4:**
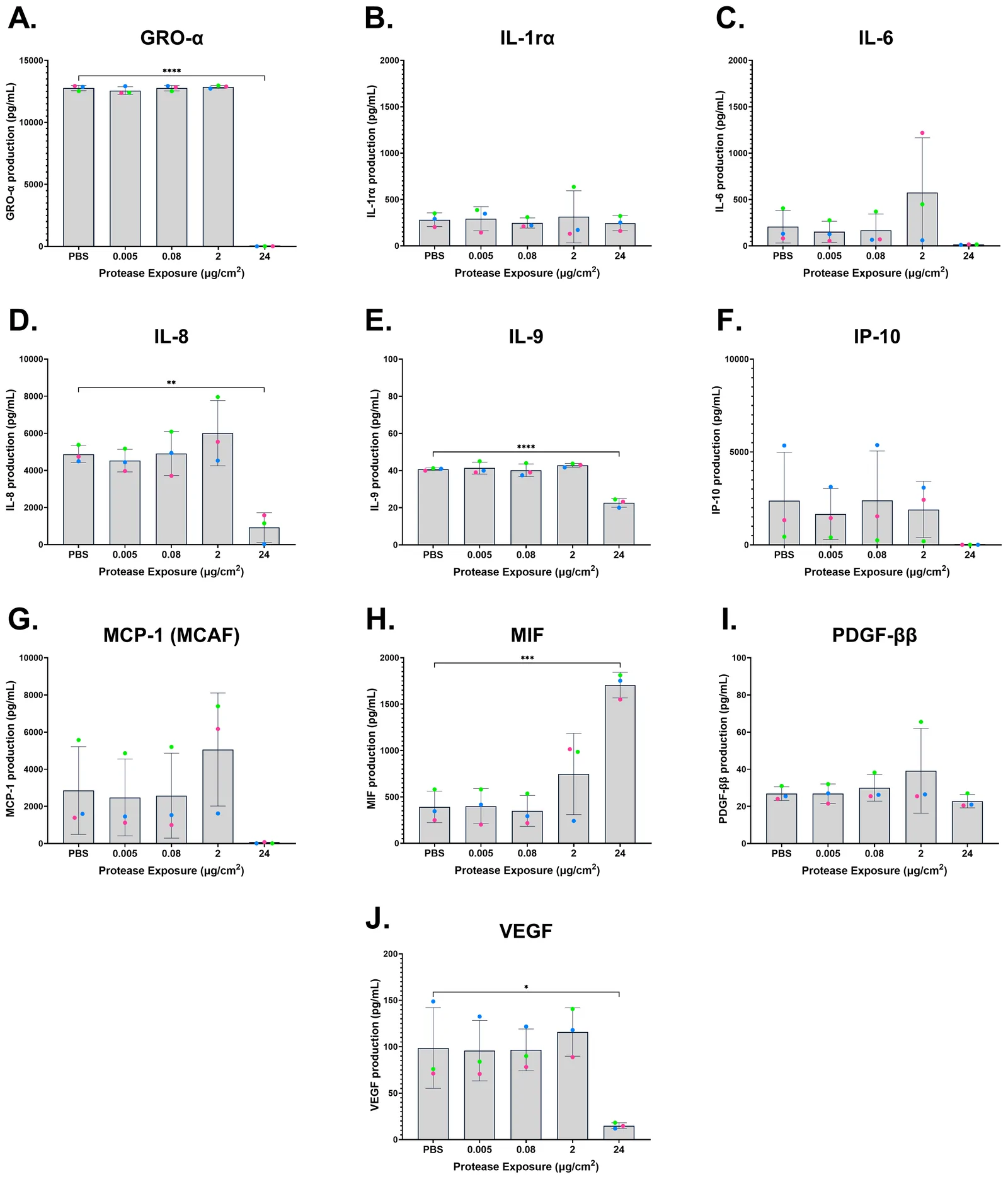
Cytokine production after protease exposures. A panel of 10 cytokines were tested on basal supernatants 24h after each protease exposure and the PBS control. **(A)** GRO-α, **(B)** IL-1rα, **(C)** IL-6, **(D)** IL-8/CXCL8, **(E)** IL-9, **(F)** IP-10, **(G)** MCP-1 (MCAF), **(H)** MIF, **(I)** PDGF-ββ, and **(J)** VEGF. An ordinary one-way ANOVA with Dunnett’s multiple comparisons was conducted for each cytokine, *p<0.05, **p<0.01, ***p<0.001, ****p<0.0001. N = 3 donors; MD0858 (green), MD0937 (pink), MD0571 (blue).

Epithelial cells produce cytokines in response to damage or other stimuli. As such, a bio-plex cytokine assay was conducted, measuring 10 cytokines in basal supernatants 24h after protease or PBS exposures; GRO-α (CXCL1), IL-1rα, IL-6, IL-8/CXCL8 (CXCL8), IL-9, IP-10, MCP-1/MCAF, MIF, PDGF-ββ, and VEGF (**Figure 4**). Technical repeat inserts for each donor were pooled together and importantly, 0.2mM PMSF was added to samples 1 hour before conducting the assays to reduce interference of any residual protease in the samples.

Notably, there were no significant changes in cytokine production after exposures of 0.005-2 µg/cm^2^ protease, compared to the PBS control. After 24 µg/cm^2^ protease exposures, there was a significant decrease in GRO-α, IL-8/CXCL8, IL-9, and VEGF cytokine production, compared to the PBS control. In contrast, there was a significant increase in MIF production after 24 µg/cm^2^ protease.

### EV characterisation

In agreement with MISEV guidelines (25), the presence of EVs from MucilAir™ inserts were first confirmed by transmission electron microscopy (TEM) and sized using nanoparticle tracking analysis (NTA) (**Supplementary Figure 2**).

**Figure 5:**
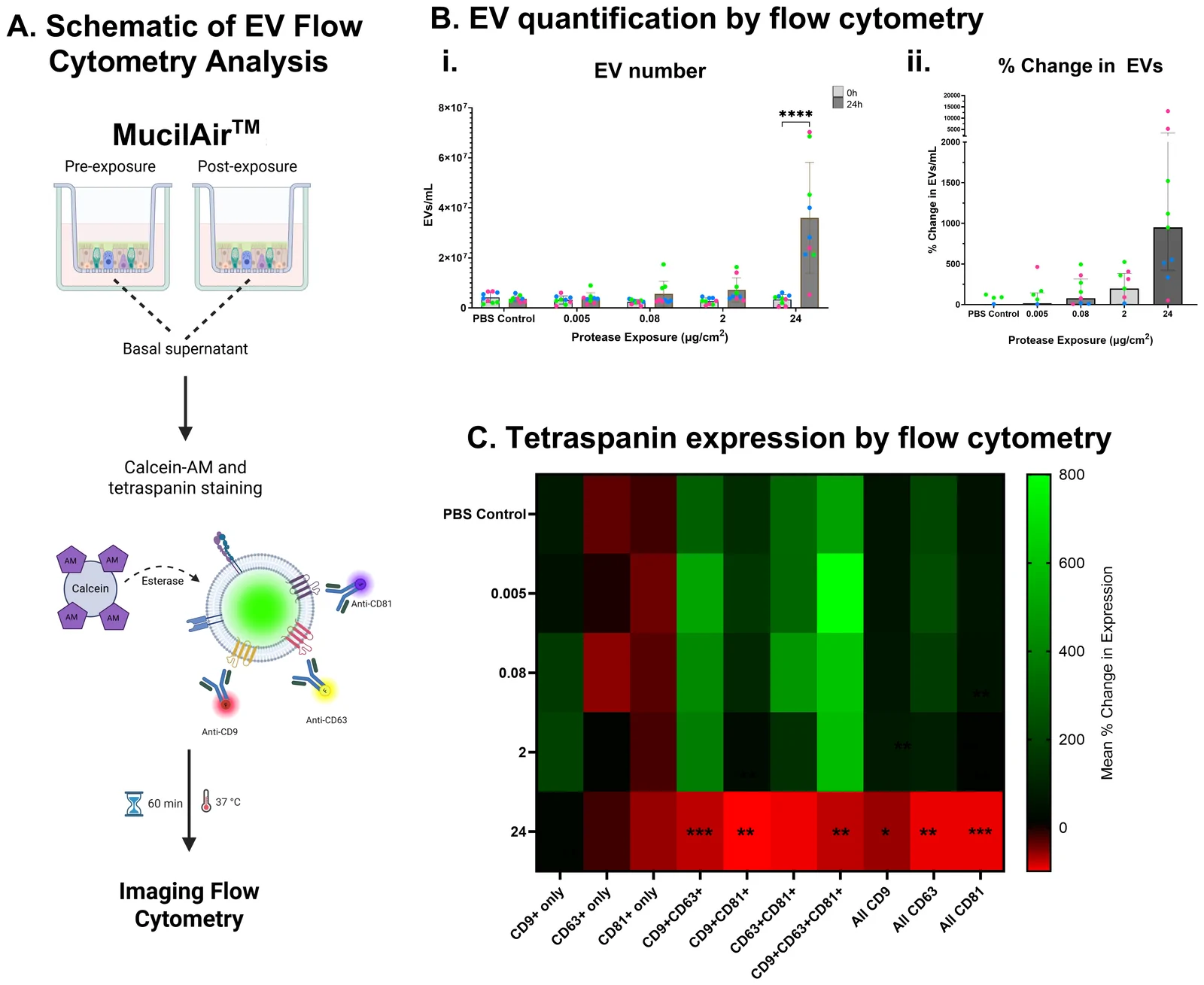
EV characterisation. **(A)** Schematic of EV flow cytometry analysis, where EVs within basal supernatants were stained with calcein-am and tetraspanins antibodies before analysis on an imaging flow cytometer. **(Bi)** The number of EVs/mL detected in samples before exposures (0h) and 24h after exposures. **(Bii)** The % change in EV production in each MucilAir insert from 0h to 24h of each exposure. **(C)** The mean % change in the number of EVs expressing each tetraspanin from 0h to 24h is presented. Different combinations of tetraspanin expressed on EVs are along the x-axis, and the exposures are along the y-axis. A mixed effects model with Sidak’s multiple comparisons was conducted for **(Bi, C)**. A repeated-measures one-way ANOVA was conducted for **(Bii)**. ns, p>0.05; *p<0.05, **p<0.01, ***p<0.001, ****p<0.0001. N = 3 donors, with 3 replicate inserts measured for each exposure. Each donor is colour coded, MD0571 (blue), MD0858 (green), MD0937 (pink).

In addition, all MucilAir™ basal supernatants, before and after exposures, were stained with calcein-AM to quantify EVs, as well as CD9, CD63, and CD81 to measure their tetraspanin expression by flow cytometry (**Figure 5A**). The results indicate a significant increase in EV production after only exposure to 24 µg/cm^2^ of protease compared to 0h (P<0.0001) (**Figure 5Bi**). Despite the increase in EVs at the highest exposure, the % change in EV number from 0h to 24h was not significantly different from the PBS control (**Figure 5Bii**).

In terms of tetraspanins expression, profiles were similar after exposures of 0-2 µg/cm^2^ of protease (**Figure 5C**). However, despite a significant increase in EV number after exposure to 24 µg/cm^2^ of protease, there was a clear decline in overall tetraspanin expression, of which most tetraspanin combinations were significantly decreased.

### EV transcriptomics analysis

**Figure 6:**
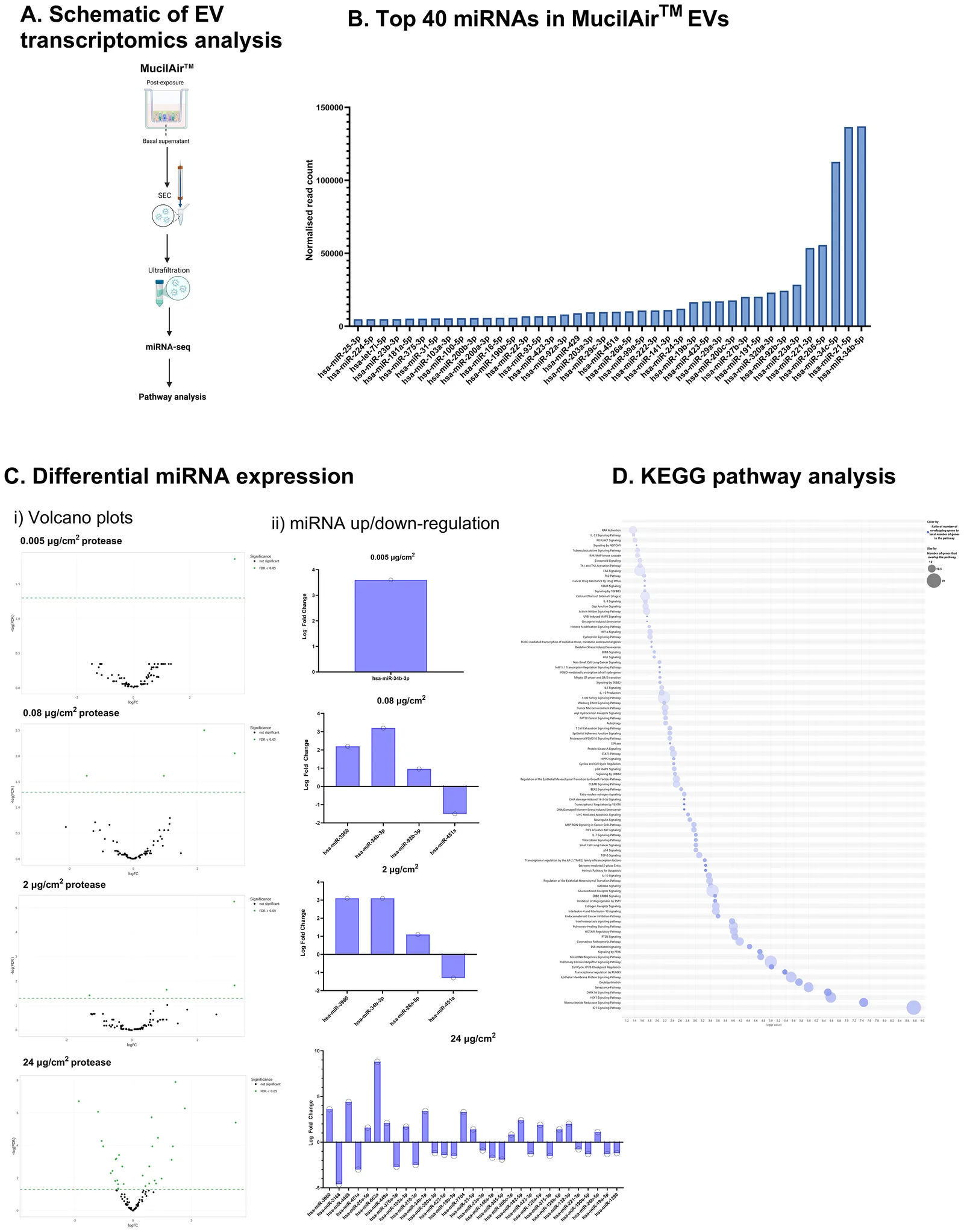
EV transcriptomics analysis. **(A)** A schematic representation of EV preparation for transcriptomics analysis, where basal supernatant samples were collected 24h after exposures and EVs were isolated by SEC and concentrated by ultrafiltration. miRNA sequencing and pathway enrichment analyses were then conducted. **(B)** The top 40 miRNAs found in MucilAir™ EVs exposed to the PBS control. **(C)** Differential miRNA expression of EVs after each exposure, when compared to the PBS control EVs; **(Ci)** volcano plots for differentially expressed miRNAs, **(Cii)** bar charts to indicate up/downregulation of the differentially expressed miRNAs. **(D)** Bubble plot categories of Ingenuity Knowledge Base (Qiagen) pathway enrichment analysis based on all significant differentially expressed miRNAs from all exposures. N = 1 donor, with 3 replicate inserts measured for each exposure.

The transcriptomic cargo in MucilAir™ EVs was examined to further clarify the molecular mechanisms by which EVs can modulate responses to nebulised protease. EVs 24 hours after exposures were SEC-isolated from the basal medium and concentrated with ultrafiltration before conducting miRNA sequencing and Qiagen IPA pathway enrichment analysis (**Figure 6A**).

There is limited research on the miRNA cargo of unstimulated primary human bronchial epithelial (HBEC)-EVs, thus, the top 40 most abundant miRNAs were first identified using EVs from the PBS control exposures (**Figure 6B**). The two most abundant were the miR-34 family, which are strongly enriched in differentiated, ciliated epithelial cells. This baseline EV cargo was also dominated by epithelial-enriched and housekeeping miRNAs, including let-7i-5p, miR-16-5p, miR-22-3p, miR-26a-5p, miR-29a/c-3p, miR-23a/b-3p, and miR-24-3p, reflecting constitutive epithelial homeostatic signalling. Several miRNAs with known roles in epithelial identity and epithelial-mesenchymal transition (EMT) regulation, particularly members of the miR-200 family and miR-205-5p were also prominent, suggesting that HBEC-EVs contribute to maintaining epithelial phenotype under resting conditions. In addition, stress- and immune-modulatory miRNAs such as miR-21-5p, miR-93-5p, miR-181a-5p, miR-221/222-3p, and miR-320a-3p were detectable at high levels, indicating that even in the absence of stimulation, HBECs export regulatory signals with potential roles in epithelial-immune communication.

The miRNA expression in PBS-exposed EVs was then compared to EVs derived after each protease exposure. The number of significantly differentially expressed miRNAs increased with increasing concentrations of protease; 1 miRNA after 0.005 µg/cm^2^ of protease, 4 miRNAs after both 0.08 and 2 µg/cm^2^ of protease, and 31 miRNAs after 24 µg/cm^2^ of protease (**Figure 6Ci**). These differentially expressed miRNAs were then analysed for their up or downregulation compared to the PBS control (**Figure 6Cii**). The significantly differentially expressed miRNAs identified after all 4 protease exposures were combined to perform pathway enrichment analysis. This identified a broad range of biological processes relevant to epithelial function and airway homeostasis. The top enriched pathways were predominantly associated with cellular signalling, inflammatory and immune regulation, cytoskeletal organisation, and cell survival and repair mechanisms. Several pathways linked to epithelial barrier maintenance, stress-response signalling, and growth factor receptor cascades also appeared among the most significantly enriched categories. In addition, pathways related to metabolic regulation and intercellular communication were represented, consistent with the potential diversity of regulatory roles attributed to EV-associated miRNAs.

### Modelling the impact of protease exposures on TEER

TEER, IL-8/CXCL8, and EV production data were then used to model the responses of the MucilAir™ to protease. The data were used to calculate the probability of TEER, IL-8/CXCL8, and EV production decreasing when exposed to protease solutions. A logistic (binary response) model was used to fit the data, and as standard, a threshold was set to P25 – 1.5 x IQR (Inter Quartile Range) to exclude outliers.

**Figure 7:**
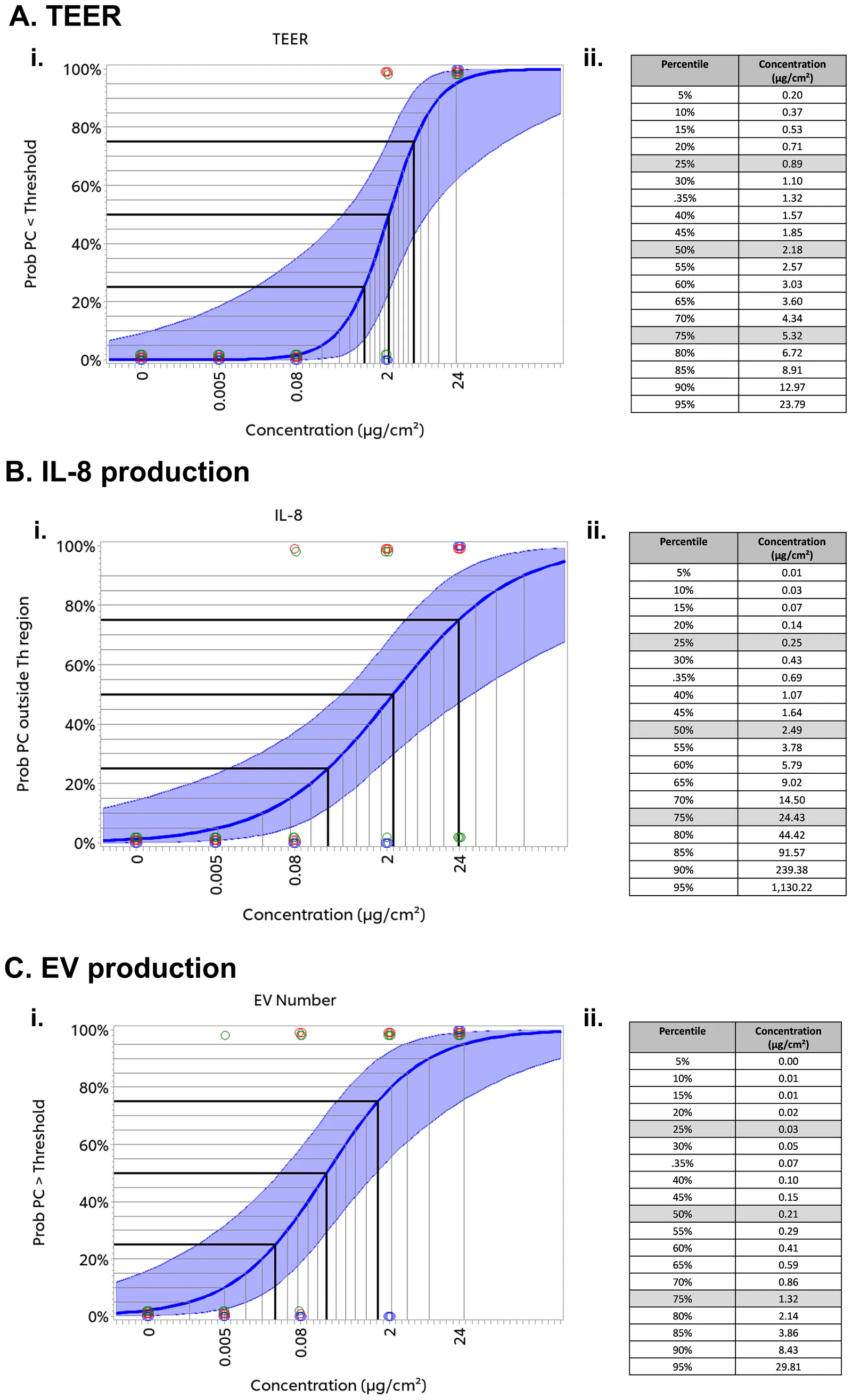
Probability modelling. A logistic (binary response) curve to model the percentage change in **(Ai)** TEER data, **(Bi)** IL-8/CXCL8 production data, and **(Ci)** EV production data. **(Aii–Cii)** A table detailing the calculated amount of protease required to disrupt TEER, IL-8/CXCL8, and EV production, at different probability percentiles. N = 3.

TEER disruption occurred predominantly at higher protease concentrations, with a 50th percentile concentration for barrier disruption estimated at 2.18 µg/cm^2^, with probabilities exceeding 90% at concentrations ≥12.97 µg/cm^2^ (**Figure 7A**).

IL-8/CXCL8 was selected for modelling because it is a well-established marker of bronchial epithelial activation and inflammation, and demonstrated a clear, dose-responsive change following protease exposure in the present study. IL-8/CXCL8 production displayed a more gradual dose-response relationship, with a 50th percentile concentration of 2.49 µg/cm^2^. High-probability inflammatory responses (>90%) were only observed at substantially higher concentrations (239 µg/cm^2^) (**Figure 7B**).

EV production was the most sensitive endpoint, with increased EV release detected at lower concentrations than TEER or IL-8/CXCL8. The 50th percentile concentration was 0.21 µg/cm^2^, and probabilities exceeded 90% at ≥8.43 µg/cm^2^ (**Figure 7C**).

Overall, logistic modelling identified EV production as the most sensitive indicator of protease exposure, preceding measurable epithelial barrier disruption and IL-8/CXCL8 release across the concentration range tested.

## Discussion

This study demonstrates a system for nebulised dosing in a human-relevant bronchial ALI model for assessing epithelial responses to inhaled proteases and provides new insight into EV-mediated signalling as a sensitive mechanistic endpoint. By combining aerosolised delivery with functional barrier measurements, cytokine profiling, and EV phenotyping and transcriptomics, we identify a threshold for epithelial disruption at approximately 2 µg/cm^2^ protease, while confirming the absence of artefactual responses associated with vehicle exposure alone, as previously observed with a liquid dosing approach. Collectively, these findings strengthen the methodological basis for incorporating ALI-based NAMs into next-generation risk assessment (NGRA) frameworks for inhaled enzymes and other airborne materials.

Firstly, this work identifies a clear distinction between liquid-based and nebulised exposure approaches. Consistent with our previous observations and those of others (11, 14), repeated apical liquid application of PBS alone resulted in reduced TEER and increased IL-8/CXCL8 secretion, indicating non-physiological epithelial stimulation attributable to the exposure method rather than the test substance itself. In contrast, nebulised PBS produced no detectable changes in barrier integrity or inflammatory signalling, confirming that aerosolised delivery better preserves epithelial homeostasis in ALI cultures. This was not a direct methodological comparison of delivery modalities, as the exposure protocols differ in several respects (including duration of apical liquid contact and liquid removal steps). Rather, they demonstrate the extent to which the repeated liquid dosing procedure itself may affect epithelial readouts and support the use of nebulisation as a less disruptive exposure approach. This is particularly relevant for NGRA, where false-positive responses arising from experimental artefacts can confound hazard identification and undermine confidence in NAM-derived data. This is supported by existing research, which shows aerosol delivery to ALI cultures more efficiently mimics real inhalation exposures with faster kinetics than adding liquid on top of cultures (26).

Despite the clear advantages of using a nebulised delivery system of protease over repeated liquid application, it does not come without challenges. Accurately characterising the dose to the cells in ALI systems is challenging because of the variability in particle deposition in ALI systems, which is influenced by the physicochemical properties of the aerosols and the type of exposure system utilized by the study (27–29). Different test substances have variable deposition rates (29), thus the deposition of each new substance tested should be determined before use, taking into account concentration of the substance, as increased viscosity with increased concentration of some substances also reduces deposition rate, due to increased droplet size (30, 31). In sedimentation aerosol exposure systems, such as the system used in this study, the aerosol droplets also tend to adhere to the inner surfaces of the chamber and the nebuliser (32), thus thorough characterisation of the settling velocity and time is essential to accurately quantify dose delivery. In addition, although MPPD modelling was used to contextualise deposited protease doses, it is important to recognise that such models rely on simplifying assumptions related to aerosol properties, airway geometry, and breathing patterns (33–35). These limitations mean that the concentrations applied here may not directly correspond to true inhalable or deposited human doses. The identified disruption threshold should therefore be interpreted as model-specific rather than as a definitive human exposure limit, highlighting the need for continued refinement of dosimetry models and quantitative *in vitro* to *in vivo* extrapolation (QIVIVE) approaches.

Taking into account these challenges with aerosolised delivery, we observed a clear concentration-dependent response to protease. Exposures of up to 0.08 µg/cm^2^ produced no measurable effects on TEER. In contrast, protease concentrations of 2 µg/cm^2^ resulted in lower TEER readings, although not significant. The highest concentration of 24 µg/cm^2^ then resulted in a significant reduction in TEER, consistent with epithelial barrier compromise. More donors and intermediate concentrations between 2-24 µg/cm^2^ protease would be beneficial in increasing power and pinpointing when TEER begins to significantly decrease.

Immunofluorescence analysis did not reveal overt disruption of tight junction proteins occludin or ZO-1 at 0-2 µg/cm^2^ of protease, and could not be measured at the highest dose of 24 µg/cm^2^ due to insert degradation. This suggests that early protease-induced barrier dysfunction may involve subtle alterations in junctional organisation and paracellular permeability that precede changes in tight junction protein levels (36), which would not be detected by confocal imaging. Alternatively, protease activity may primarily affect non-junctional components, such as surface mucins and cilia motility, leading to functional impairment prior to overt structural degradation (37, 38).

In addition, 10 cytokines were measured in basal supernatants and exposure to 24 µg/cm^2^ protease resulted in reduced GRO-α, IL-8, IL-9, and VEGF concentrations, alongside increased MIF production. GRO-α and IL-8 are important mediators of neutrophil recruitment (39), while VEGF is associated with tissue repair and epithelial remodelling (40). These reductions therefore suggest that protease exposure may alter epithelial signalling pathways involved in inflammatory cell recruitment and tissue homeostasis. Conversely, the increase in MIF indicates activation of specific pro-inflammatory pathways. The simultaneous suppression of several cytokines together with the induction of MIF suggests a selective modulation of epithelial responses rather than a general increase or decrease in inflammation. LDH measurements also did not show significant increases even at the highest protease concentration, despite visible epithelial degradation. This apparent discrepancy likely reflects proteolytic degradation of LDH during the 24-hour incubation period (41), highlighting an important limitation of conventional cytotoxicity assays when assessing proteolytically active substances. Alternative viability assays could include ATP-based measurements or resazurin solutions.

A major strength of this study is the integration of EV analysis as a mechanistically informative endpoint. While EV numbers remained stable across PBS and low protease exposures, a pronounced increase in EV release was observed at 24 µg/cm^2^, coinciding with severe epithelial barrier disruption. Notably, as we saw in previous work (11), this increase in EV abundance was accompanied by a reduction in tetraspanin expression, suggesting a shift in EV biogenesis pathways or the release of heterogeneous vesicle populations associated with cellular stress, injury, or membrane fragmentation. These findings support the concept that EV quantity and surface marker composition provide complementary information on epithelial state, distinguishing adaptive or sub-threshold responses from overt injury.

With limited existing data (42), we identify the most abundant miRNA in unstimulated MucilAir™-derived EVs, with the top three including miR-34b-5p, miR-34c-5p, and miR-21-5p. The unstimulated EVs contained a rich repertoire of miRNAs associated with epithelial identity, homeostasis, and immune modulation, consistent with emerging evidence that airway epithelial EVs play active roles in maintaining tissue equilibrium (22, 23). EV transcriptomic analysis further demonstrated the sensitivity of EV cargo to protease exposure. The number of differentially expressed EV-miRNAs increased with protease concentration, with minimal changes at low doses and extensive reprogramming at 24 µg/cm^2^. Pathway enrichment analysis implicated signalling pathways involved in inflammation, cytoskeletal organisation, epithelial repair, and stress-response signalling, aligning closely with known key events in adverse outcome pathways for respiratory toxicity (2). Importantly, several of these miRNA-regulated pathways are linked to barrier maintenance and immune-epithelial crosstalk, supporting the hypothesis that EV-miRNAs may act as early propagators of epithelial stress signals prior to overt tissue damage. However, it must be noted that these EVs isolated from the highest protease concentration exposure may originate predominantly from damaged, apoptotic or necrotic cells. Consequently, the biological relevance of the EV and miRNA profile at this concentration may differ from that observed at lower concentrations and should be interpreted as a response associated with extensive tissue injury rather than an early adaptive cellular response. But overall, these data highlight EV-miRNA profiling as a powerful, non-invasive readout that captures early molecular perturbations not detectable by conventional functional assays alone. Incorporating such endpoints into NAM-based testing strategies could enhance sensitivity and mechanistic resolution.

Further statistical and modelling analysis was conducted by fitting a binary response model to the TEER, IL-8/CXCL8, and EV production data. Modelling MucilAir™ responses to nebulised protease revealed differential sensitivity across endpoints. EV release was the most sensitive, with a median effective concentration of 0.21 µg/cm^2^ and >90% probability at 8.43 µg/cm^2^ of protease, suggesting it as an early indicator of epithelial stress. This was closely followed by TEER measurements, which had a >90% probability of barrier loss at ≥12.97 µg/cm^2^. IL-8/CXCL8 production showed a gradual dose-response, with >90% probability inflammatory activation observed only at substantially higher exposures (≥239 µg/cm^2^). These findings highlight that EV release precedes barrier disruption and cytokine signalling and demonstrate the utility of probabilistic modelling for predicting epithelial responses to protease exposure *in vitro*. A limitation of the current model is the absence of an immune component, meaning that identified disruption thresholds are based solely on epithelial responses. Future studies incorporating immune cell co-cultures or more complex airway microphysiological systems could improve the ability of the model to capture the cellular interactions that occur *in vivo* and enhance confidence in human-relevant predictions.

To contextualise the different responses observed in the present study, and as discussed further in more detail in our previous study (11), the introduction of exposure controls and occupational exposure limits (OELs) for enzymes used in detergent manufacturing has minimised sensitisation and prevented symptoms (10, 43). In addition, a benchmarking approach underpins the risk assessment of enzyme-containing consumer products, drawing on derived minimal effect levels (DMELs). Previously, our study using the MPPD model with refinements (11), utilised protease concentrations covering those associated with a low to high risk of allergic sensitisation in an occupational setting (8 h exposure to <1–10,000 ng protease per m^3^), which extrapolated to tracheobronchial tissue doses of <0.03–278 pg per cm^2^. The protease concentrations used in the present study exceeded estimated occupational exposure levels associated with sensitisation risk, but were included to identify the threshold at which epithelial disruption occurs in this model. These findings suggest that biologically meaningful epithelial disruption occurs only at exposure levels substantially higher than those typically encountered in well-controlled occupational settings.

Overall, this study demonstrates that nebulised delivery to human bronchial ALI cultures provides a physiologically relevant, artefact-free approach for assessing protease-induced epithelial stress. Through modelling responses, we identify TEER, EV, and IL-8/CXCL8 disruption thresholds at differing concentrations of protease. EV analysis further enhances mechanistic insight, with protease exposure induces dose-dependent reprogramming of EV-miRNA cargo which could affect barrier, stress, and immune signalling pathways. While nebulised delivery better preserves epithelial homeostasis than repeated liquid dosing, accurate dose quantification remains challenging due to aerosol deposition variability and model-specific assumptions, highlighting the need for continued refinement of inhalation dosimetry and QIVIVE approaches. In conclusion, this work advances the methodological and biological foundations for using ALI-based NAMs in respiratory risk assessment and positions EV profiling as a promising mechanistic biomarker for early epithelial disruption following inhaled protease exposure.

## Data Availability

The data presented in the study are deposited in the NCBI SRA repository, accession number PRJNA1506939.
